# Supplementary material for: Multi-photon polymerization using upconversion nanoparticles for tunable feature-size printing
Source: Nanophotonics. 2023 Jan 10;12(8):1527–36. doi: 10.1515/nanoph-2022-0598 (PMC11501605; doi:10.1515/nanoph-2022-0598)
Supplement: Supplementary file 1 — Supplementary Material Details [file j_nanoph-2022-0598_suppl.pdf]

Supplementary Information

[2]Qianyi Zhang, [2]Antoine Boniface, [1]Virendra K. Parashar, [1]Martin A. M. Gijs, [2]Christophe Moser

[1]Laboratory of Microsystems LMIS2, School of Engineering, Institute of Electrical and Micro Engineering, Ecole Polytechnique Fédérale de Lausanne, Lausanne, Switzerland

[2]Laboratory of Applied Photonics Device, School of Engineering, Institute of Electrical and Micro Engineering, Ecole Polytechnique Fédérale de Lausanne, Lausanne, Switzerland

1. Voxel size versus NIR dose

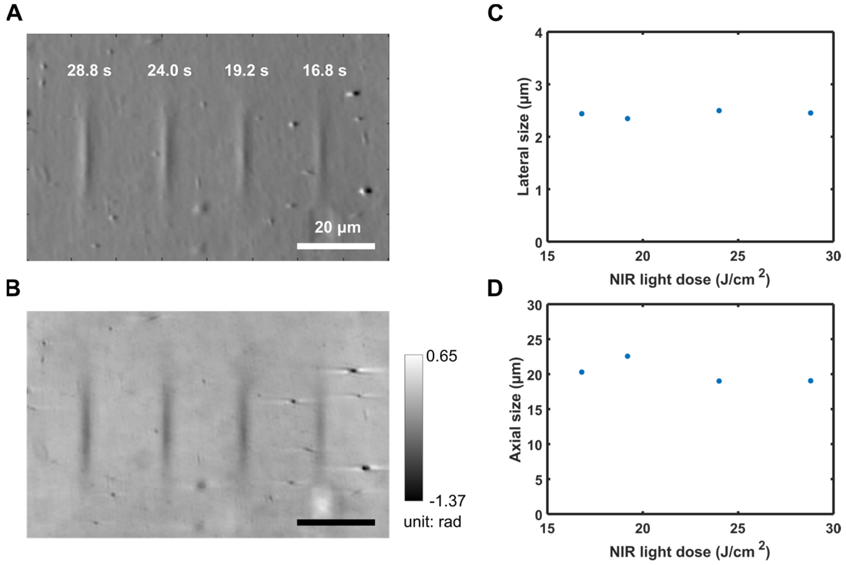

Figure S1
(A) DPC image of voxels with different excitation time at the NIR intensity of 1.1×10^5 W/cm^2 and (B) reconstructed phase of voxels printed. Lateral (C) and axial (D) size of the printed voxels versus the light dose.

2. NIR and fluorescence beam profile

The beam profiles of the NIR light and the upconverted fluorescence are measured by the setup in Figure S2B to understand how the tunable feature size is achieved in UCNPs-assisted 3D printing. The capillary is filled with gels containing UCNPs (10 mg/mL) and gelatin (15 wt%). The position of the capillary is adjusted so that the focal spot is in the center of the capillary, minimizing the influence of the glass wall on the recorded beam profile. The objective MO_3 is moved along the optical axis (z-axis) by a motorized stage and the transverse intensity profile at each *xy*-plane is recorded by a camera. The fluorescent profile of each intensity is measured at the same position of the capillary. The measurement is performed in three capillaries. Figures S2A and B show the longitudinal sections of the normalized beam profile. The NIR beam profile displays a gaussian beam with spherical aberration. Its beam size and shape do not change with the laser power. The fluorescence from UCNPs, however, has different beam profiles with the increase of the excitation intensity. For comparison, all the intensities of the fluorescence are normalized by the intensity range of the fluorescence at a NIR intensity of 8 kW/cm^2.

For example, the peak intensity of fluorescence at 8 kW/cm^2 is 1, and the peak intensity of fluorescence at 30 kW/cm^2 is 7, larger than the increase of the NIR intensity.

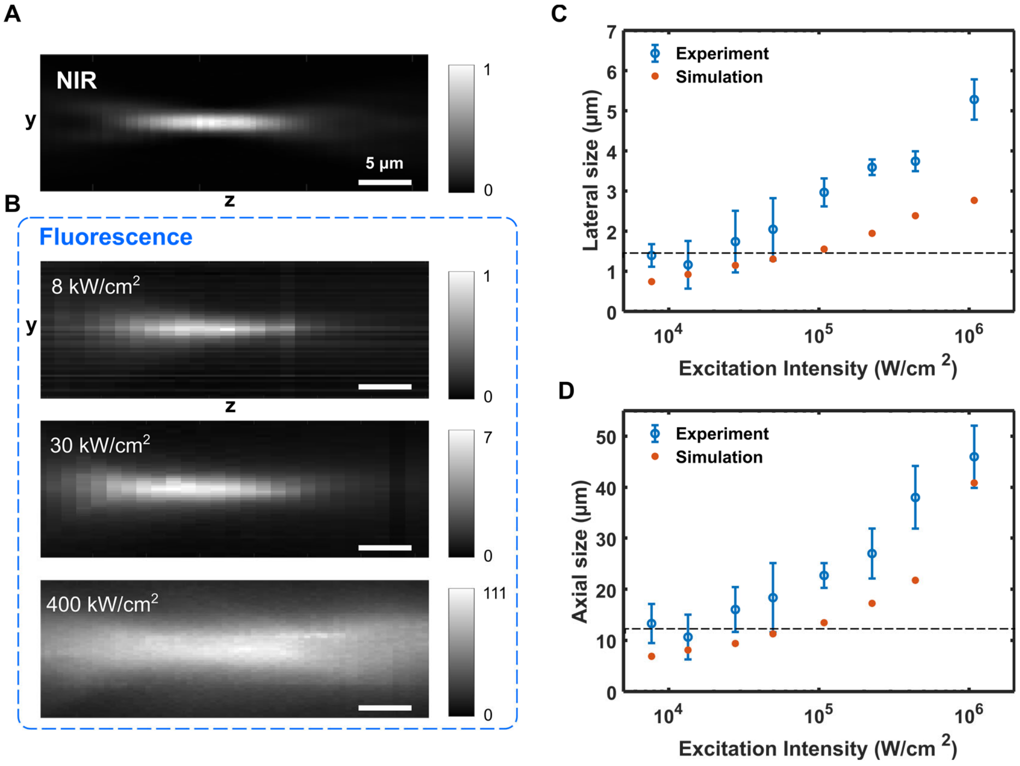

Figure S2:
The beam profile of the NIR light (A) and fluorescence at the NIR excitation intensity of 8, 30, 400 kW/cm^2. Lateral (C) and axial (D) size of the fluorescence voxel versus NIR excitation intensity. The dotted line marks the focal spot size of the NIR beam in each direction.

The lateral and axial size of the beam is defined as the FWHM of the intensity profile across the center in corresponding directions. The simulation of the fluorescent profile is based on the NIR beam profile and the power-dependent emission curve. Each pixel is assigned to a fluorescence intensity according to its NIR intensity. Then the lateral and axial size of the simulated profile is measured in the same way as that of experimental data. The discrepancies in the spot size of the experiment and the simulation are similar to those of fluorescence and printing, indicating that it is mainly caused by the way that the fluorescence profile is measured.

3. Setups in this study

Two different NIR beam sizes are used in this study by changing the collimator. In the measurement of emission spectra of UCNPs, a beam with constant NIR intensity (low divergence) within the gels is needed because of the power-dependent nonlinearity of the fluorescence. Therefore, a continuous-wave laser at 976 nm (900 mW, BL976-PAG900, Thorlabs) with a Polarization-Maintaining (PM) optical fiber is collimated by a lens (F230APC-980, Thorlabs, f = 4.55 mm). As the beam (d = 1 mm) does not fill the aperture of the back focal plane of the objective MO_1 (M Plan Apo NIR 20X, Mitutoyo), the

focused beam has a beam waist of 11 mm (FWHM) with a low numerical aperture (NA ~ 0.05). The beam size is almost unchanged within the size of the capillary, providing a constant NIR intensity as well as a large volume of illumination. For printing, however, a focused beam with higher NA is preferred to have a high spatial resolution. Therefore, a large beam collimator (Thorlabs F810APC-1064) is coupled to the PM optical fiber to generate an 8-mm beam filling the whole aperture of MO_1. The focused beam has a diameter of 1.44 mm in the gels. The setup for measuring the beam profile also applies this optical configuration to calibrate the real beam size during the printing. The NIR intensity is tuned by modulating the current and applying different density filters (not illustrated in the figure) before MO_1.

Figure S3A shows the setup for emission spectra measurement. After being coupled by the collimator, the laser beam is focused by a MO_1 into a glass square capillary (100 μm ×100 μm, CM Scientific). The capillary is filled with gels containing UCNPs and gelatin to mimic the environment during the printing but without a chemical reaction. The fluorescence signal is then collected by a second objective MO_2 (UApo/340 20x, Olympus) in an orthogonal direction and coupled by a lens (LA4052-A-ML, Thorlabs) into a multi-mode fiber (QP400-025-SR/BX, Ocean Insight) and recorded by a spectrometer (Ocean Optics USB4000, Ocean Insight).

Figure S3B illustrates the setup for NIR and fluorescent beam profile measurement. The capillary is also filled with UCNPs in gelatin solution. The intensity beam profile is imaged by a microscope objective MO_3 (LIO-60X, Newport) and a lens (Thorlabs AC254-200-A-ML f = 200 mm) onto a camera (Basler acA1300-30gm). The objective is mounted on a motorized stage with a piezo linear actuator (Picomotor 8303, Newport) to achieve a sub-micro step size between each plane.

Figure S3C shows the setup for 3D printing. The capillary is filled with resin containing UCNP-LAP (10 mg/mL) and gelMA (15 wt%). A red light-emitting diode (LED) is applied as the illumination light source to provide a live view of the printing process in two orthogonal directions (bright-field imaging). The front view (*xy*-plane) at the center of the capillary is imaged via MO_1 and a lens (Thorlabs AC254-200-A-ML f = 200 mm) onto Cam 2 (Basler acA1300-30gm). NIR light is filtered by a dichroic mirror (DM, FF699-FDi01-t1-25x36, Semrock) and a band-pass (BP) filter (center wavelength = 633 mm). The side view (*yz*-plane) is collected via MO_4 (Olympus Plan N 20X) and a lens (f = 200 mm) and recorded by Cam 3. The NIR light is cut off by a short-pass (SP) filter (FF01-720/SP-25, Semrock).

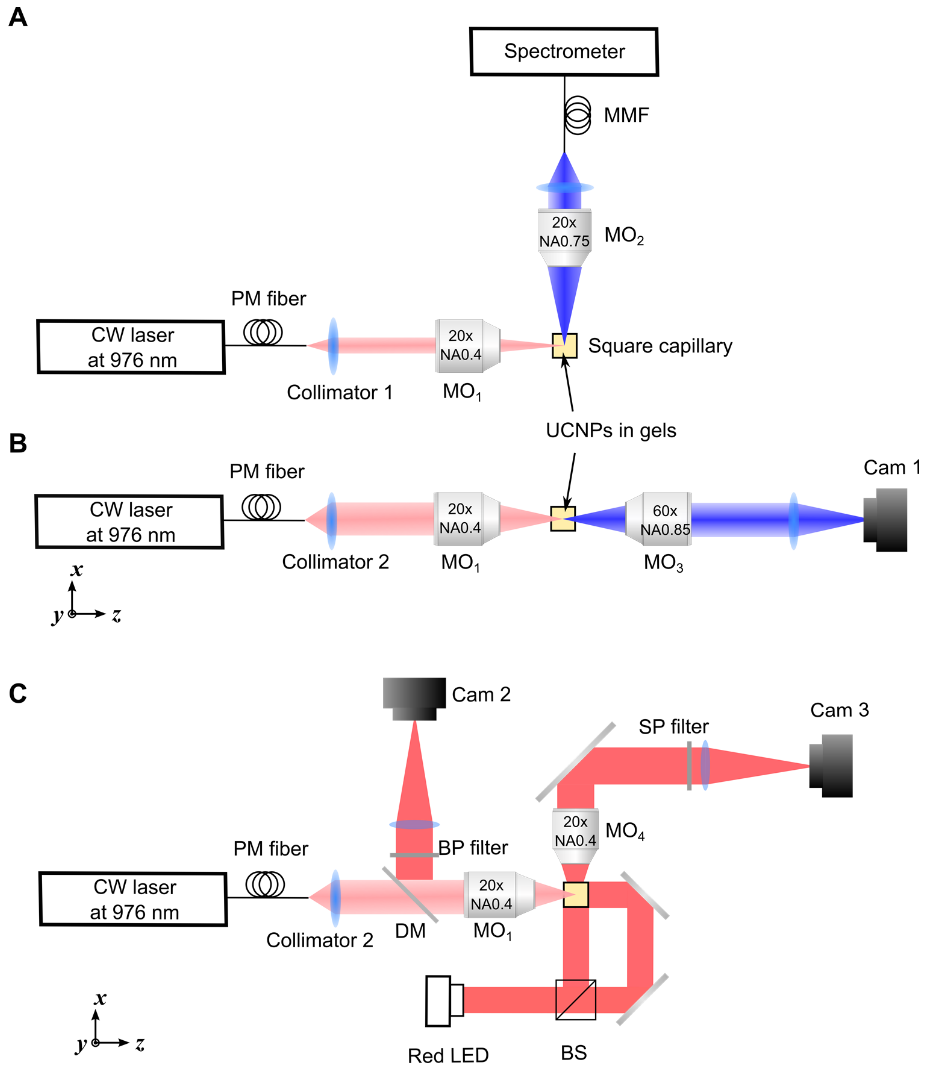

Figure S3:
Experimental setup. (A) Measuring the emission spectra of UCNPs. (B) Characterization of NIR and fluorescent beam profile. (C) 3D printing.

4. Synthesis

Materials

Yttrium(III) acetate hydrate (99.9%), ytterbium(III) acetate hydrate (99.99+%), Thulium(III) acetate hydrate (99.9%), sodium hydroxide (reagent grade), ammonium fluoride (reagent grade), tech grade oleic acid (90%), tech grade 1-octadecene (90%), methanol, ethanol (anhydrous), cyclohexanes, hydrochloric acid (37% ultrapure), Dimethyl phenylphosphonite, 2,4,6-trimethylbenzoyl chloride, lithium bromide, 2-butanone, were purchased from Merck & Co (Sigma-Aldrich). All the solvents were deoxygenated and degassed using multiple cycles of vacuum/freeze/thawing under an argon gas atmosphere. All the syntheses were carried out under the flow of argon gas.

Synthesis of $NaYF_4$:Yb/Tm core UCNPs (0.5 mol% Tm^{3+}, 30 mol% Yb^{3+} doped)

In a typical synthesis, Yttrium (III) acetate hydrate (0.556 mmol), ytterbium (III) acetate hydrate (0.240 mmol), and Thulium (III) acetate hydrate (0.004 mmol) were added to a 100 ml 3 neck Schlenk flask containing oleic acid (6 ml) and 1-octadecene (15 ml) and heated to 140 C under vacuum having argon atmosphere for 90 min and cooled to 50 °C. To this, methanol solution (10 ml) of ammonium fluoride (3.2 mmol) and sodium hydroxide (2 mmol) was added dropwise and stirred for 30 min. The reaction vessel was then heated to 70 °C to remove methanol and subsequently heated to 300 °C (~10 °C/min) under argon and maintained for 60 min. The reaction mixture was then cooled to room temperature and the NCs were precipitated by the addition of ethanol, collected by centrifugation, and redispersed in cyclohexane. Repeating this process thrice, before using them as core UCNPs in the next step.

Synthesis of $NaYF_4$ shell precursor

Yttrium (III) acetate hydrate (0.8 mmol), was added to a 100 ml three-neck Schlenk flask containing oleic acid (6 ml) and 1-octadecene (15 ml) and heated to 140 °C under a vacuum argon atmosphere for 90 min and cooled to 50 °C. A methanol solution (10 ml) of ammonium fluoride (4 mmol) and sodium hydroxide (2.5 mmol) was added dropwise to this mixture and under continuous stirring for 30 min. The reaction vessel was then heated to 70 °C to remove methanol under argon gas and maintained for 60 min. The reaction mixture was then cooled to room temperature and used as a shell precursor.

Synthesis of $NaYF_4$:Yb/Tm @ $NaYF_4$ core-shell UCNPs

Layer-by-layer successive epitaxial shell growth of $NaYF_4$ was achieved on $NaYF_4$:Yb/Tm core UCNPs. Core UCNPs were added to 1-octadecene (5ml) in a 3-neck Schlenk flask and heated to 300 °C in an argon atmosphere. To this, shell precursor solution was injected @ 5 µL/sec using a nemesys syringe pump system. The ripening was done at 300 °C for 30 min. After ripening the solution was cooled down to room temperature and the core-shell NCs were precipitated and washed as outlined for core UCNPs and finally dispersed in hexane (5 ml).

Preparation of ligand free $NaYF_4$:Yb/Tm @ $NaYF_4$ core-shell UCNPs

The ligand-free core-shell UCNPs were prepared through the acidic treatment of the OA-capped UCNPs. In a Teflon flask, 5 mL 2M hydrochloric acid was added to 100 mg of the core-shell UCNPs, dispersed in hexane (20 mg/mL) to protonate the oleate ligands, leading to the removal of the oleic acid from the UCNPs' surface. This mixture was vigorously stirred at room temperature for 15 min and left unhindered. The organic solvent phase became transparent. The mixture was then ultrasonicated for 30 min and centrifuge at 9000 rpm for 15 min and washed with a mixture of water and ethanol (in the ratio of 1:1) twice to discard the organic layer containing the oleic acid molecules. The free UCNPs were dispersed in distilled water and stored at 4 °C.

Synthesis of LAP photoinitiator

LAP photoinitiator was synthesized in a two-step process. First, Dimethyl phenylphosphonite was reacted with 2,4,6-trimethylbenzoyl chloride via a Michaelis-Arbuzov reaction. At room temperature and under an argon atmosphere, 2,4,6-trimethylbenzoyl chloride (0.018 mol) was added dropwise to an equimolar amount of continuously stirred dimethyl phenylphosphonite. The reaction mixture was stirred for 18-24 hours. To this, lithium bromide (6.1 g, at least four times the amount) in 100 mL of 2-butanone was added to the reaction mixture and heated to 50 °C. After 10-15 minutes, a solid precipitate formed. The mixture was cooled to room temperature, allowed to rest for four hours, and filtered. The filtrate was washed and filtered 3 times with 2-butanone to remove unreacted lithium bromide. Excess solvent was removed by vacuum under a dark inert atmosphere. The product, lithium phenyl-2,4,6-trimethylbenzoylphosphinate (lithium acylphosphinate- LAP).

Preparation of $NaYF_4$:Yb/Tm @ $NaYF_4$ core-shell UCNPs @ LAP photoinitiators

UCNPs@LAP adduct was prepared by coating UCNPs (positive) and LAP (negative) through electrostatic interactions. A solution of LAP (200 mg) dissolved in Millipore deionized water was added dropwise to an aqueous solution containing 200 mg of UCNPs under ultrasonication, and the resulting mixture was further sonicated for 5-7 hours. The purified UCNP@LAP was obtained followed by centrifugation and further dispersion in water before use.

Synthesis of gelMA

10 g of gelatin type A (Sigma-Aldrich) was dissolved in 100 mL of phosphate-buffered saline (PBS) at 55 °C for 30 min. Then 8 mL of methacrylic anhydride (Sigma-Aldrich) was added dropwise (0.5 mL/min) and the mixture was left under stirring at 50 °C for 3 hours, followed by removal of unreacted anhydride by centrifugation at 3000 rpm for 10 min and dialysis (cellulose membrane with weight cut-off of 12–14 kDa, Sigma Aldrich) at 40 °C for 4 days against demineralized water and lyophilized. To prepare the resin for printing, 15 mg of gelMA were dissolved in 80 μL of water at 40 °C for 30 min and 20 μL of NP-LAP aqueous solution (50 mg/mL) was mixed to form a final concentration of 10 mg/mL NP-LAP and 15 wt% gelMA. A high concentration of gelMA was used to minimize the flow of the resin caused by gravity. The resin was stored at 4 °C until further use.
